# The effect of 24-week belimumab treatment withdrawal followed by treatment restart in patients with SLE: an open-label, non-randomised 52-week study

**DOI:** 10.1186/s13075-022-02723-y

**Published:** 2022-02-16

**Authors:** Sang-Cheol Bae, Damon L. Bass, Myron Chu, Paula Curtis, Richard Dimelow, Laurence Harvey, Beulah Ji, Regina Kurrasch, Saima Muzaffar, Raj Punwaney, David A. Roth, Yeong-Wook Song, Wendy Xie, Fengchun Zhang

**Affiliations:** 1 Department of Rheumatology, Hanyang University Hospital for Rheumatic Diseases and Hanyang University Institute for Rheumatology Research, Seoul, Republic of Korea; 2 Immunoinflammation, GlaxoSmithKline, Collegeville, PA USA; 3 Department of Development Biostatistics, GlaxoSmithKline, Brentford, Middlesex UK; 4 Clinical Pharmacology, GlaxoSmithKline, Stevenage, Hertfordshire UK; 5 Immunoinflammation R&D, GlaxoSmithKline, Brentford, Middlesex UK; 6 Clinical Development, GlaxoSmithKline, Gunnels Wood Road, Stevenage, Hertfordshire SG1 2NY UK; 7 Division of Rheumatology, Seoul National University Hospital, Seoul National University, Seoul, Republic of Korea; 8 Department of Rheumatology and Clinical Immunology, Peking Union Medical College Hospital, Chinese Academy of Medical Sciences and Peking Union Medical College, Beijing, China

**Keywords:** Systemic lupus erythematosus and autoimmunity, B cells, Lymphocytes, Biological therapies, Biomarkers

## Abstract

**Background:**

Treatment goals for patients with systemic lupus erythematosus (SLE) include minimising disease activity and reducing the risk of flares. Although belimumab is effective at reducing disease activity and risk of severe flares, it was previously unknown what the clinical effects were upon treatment discontinuation. The objective of this study was to assess the impact of temporary withdrawal of intravenous (IV) belimumab in patients with SLE.

**Methods:**

This multicentre, open-label, non-randomised, 52-week study (GSK Study BEL116027; NCT02119156) recruited patients with SLE and stable low disease activity, of whom those on belimumab 10 mg/kg IV plus standard therapy either discontinued belimumab for 24 weeks and then restarted belimumab 10 mg/kg IV every 4 weeks (q4w) for 28 weeks (treatment holiday [TH] group), or continued on belimumab 10 mg/kg IV plus standard therapy q4w for 52 weeks (treatment continuation [TC] group). The primary endpoint was median time to first Safety of Estrogens in Lupus Erythematosus National Assessment-SLE Disease Activity Index (SELENA-SLEDAI) Flare Index flare. Secondary and other endpoints included rate of any flare, time to severe flare, time to renal flare and rebound (SELENA-SLEDAI score exceeding parent study baseline). Data on rebound phenomenon in patients with any disease level of SLE who had permanently withdrawn from further belimumab treatment (long-term discontinuation group [LTD]) were also assessed. Safety was assessed.

**Results:**

The primary endpoint was not evaluable in the TH (*n* = 12) and TC (*n* = 29) groups as fewer than half of patients flared. Unadjusted flare rates per patient-year were 1.0 during treatment discontinuation and 0.3 during treatment restart (0.6 overall) in the TH group and 0.6 in the TC group; there were no severe or renal flares. No TH patients rebounded; 2 (6.9%) TC patients rebounded; 2 (5.1%) patients in the LTD group rebounded. There were no new safety signals.

**Conclusions:**

Twenty-four-week belimumab discontinuation did not appear to increase the risk of flares or rebound in patients with low SLE disease activity; flare rates were low in both groups. Further studies may help to fully determine the effect of belimumab discontinuation.

**Trial registration:**

ClinicalTrials.gov, NCT02119156. Registered on April 21, 2014.

**Supplementary Information:**

The online version contains supplementary material available at 10.1186/s13075-022-02723-y.

## Background

Systemic lupus erythematosus (SLE) is a chronic, multisystem autoimmune disease characterised by autoantibody production and abnormal B cell function [1]. Patients with SLE experience heterogeneous clinical manifestations, chronic inflammation and a relapsing and remitting disease pattern consisting of SLE flares alternating with periods of less severe, but persistent, disease activity [2, 3]. Flares pose a substantial burden in SLE and are associated with increased disease activity, long-term organ damage and considerable healthcare costs [3, 4]. As such, SLE treatment goals include minimising disease activity and reducing the risk of flares [5].

Belimumab is a B-lymphocyte stimulator (BLyS)-specific inhibitor approved as an add-on therapy to treat active, autoantibody-positive SLE [6]. This biologic prevents BLyS from binding to receptors on B cells, thereby inhibiting B cell survival and differentiation into immunoglobulin (Ig)-producing plasma cells [7, 8]. This action is associated with a reduction in SLE disease activity and the risk of severe flares, as established in four phase 3, randomised, placebo-controlled trials [9–12].

Data on the effect of temporary belimumab withdrawal (‘treatment holiday’ [TH]) and the potential for rebound phenomenon are important to inform clinical management of patients with SLE, as some patients may need temporary treatment discontinuation. In determining the impact of restarting belimumab treatment after a temporary withdrawal, it is also of clinical importance to assess the risk for hypersensitivity reactions. However, it was previously unknown what the clinical effects of belimumab discontinuation were. A case series has suggested a possible rebound effect in three patients following belimumab discontinuation that may be due to an increase in BLyS levels leading to disease flares [13].

This study was designed to investigate the efficacy and safety of temporary discontinuation of belimumab, compared with treatment continuation (TC), in patients with SLE and stable low disease activity. Data on rebound phenomenon in patients with any disease level of SLE who had permanently withdrawn from further belimumab treatment (long-term discontinuation group, LTD) were also assessed.

## Methods

### Study design

**Fig. 1:**
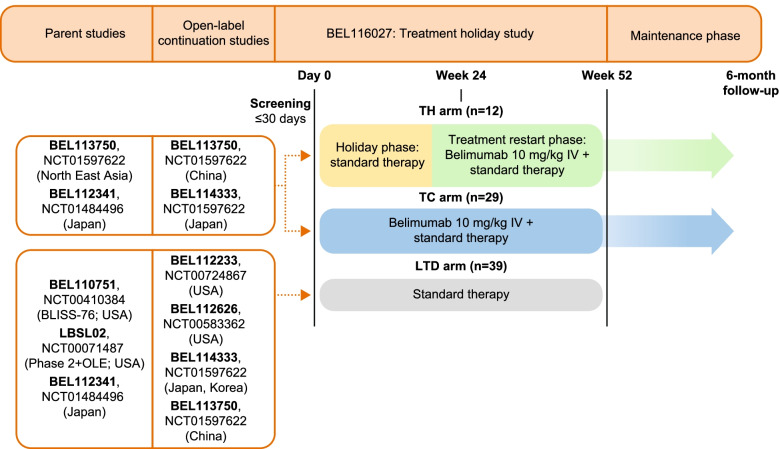
Study design. BLISS, Study of Belimumab in Subjects with SLE; LTD, long-term discontinuation; OLE, open-label extension; TC, treatment continuation; TH, treatment holiday

This multicentre, open-label, non-randomised, 52-week study (GSK Study BEL116027; NCT02119156) conducted across 20 sites in China, Japan, Korea and the USA investigated the effect of temporary (24 weeks) belimumab discontinuation followed by 28-week re-introduction of belimumab treatment in adult patients with SLE and stable low disease activity (defined below), compared with uninterrupted belimumab treatment (Fig. 1). Patients were recruited from four open-label continuation studies (GSK Study BEL113750, NCT01597622; BEL114333, NCT01597622; BEL112233, NCT00724867; and BEL112626, NCT00583362).

Patients completed a ≤ 30-day screening period before entering a 52-week treatment/observation phase during which patients received either (i) standard therapy (steroids, immunosuppressants, antimalarials, non-steroidal anti-inflammatory drugs) only for the first 24 weeks of the study, then restarted belimumab 10 mg/kg IV treatment every 4 weeks (q4w) plus standard therapy for a further 28 weeks (TH group) or (ii) belimumab 10 mg/kg IV q4w plus standard therapy for the entire 52-week period (TC group). A long-term discontinuation (LTD) group, in which patients with any level of SLE disease activity received standard therapy only for the entire 52-week period, was also included as an additional control to assess rebound phenomenon in patients expected to remain off belimumab therapy for ≥ 12 months. Standard therapy, including steroid use or dose changes, was at the discretion of the investigator. During the treatment/observation phase, all patients attended q4w clinic visits for the assessment of efficacy and safety.

At the end of the 52-week period, patients in the TH and TC groups had the option to enter a maintenance phase, during which they continued on belimumab until either they switched to commercially available belimumab, were transferred to an active protocol to continue receiving belimumab or the study was terminated. Patients in the TH group whose condition worsened during the 24-week standard-therapy-only phase could restart belimumab therapy early and enter the maintenance phase. In the TH and TC groups, a follow-up visit was scheduled at 16 weeks after withdrawal from the study or last belimumab dose; those patients who completed the study also attended a 6-month follow-up visit.

### Study population

Eligible patients were ≥ 18 years of age and had received belimumab 10 mg/kg IV for ≥ 6 months during one of the open-label continuation studies. Eligibility for the TH and TC groups required low disease activity, defined as a Safety of Estrogens in Lupus Erythematosus National Assessment-SLE Disease Activity Index (SELENA-SLEDAI) score ≤ 3 after ≥ 6 months of belimumab treatment, complement (C3 and C4) levels ≥ lower limit of normal and stable SLE treatment during the 30-day screening period. Additionally, patients in the TC group continued to receive belimumab 10 mg/kg IV every 4 weeks (q4w) and received the first dose of IV belimumab in the current study 2–8 weeks after their last dose in their previous extension study. Patients in the LTD group could have had any level of SLE disease activity, must have withdrawn from belimumab treatment ≤ 8 weeks prior to entry into the current study, and intended to remain off belimumab therapy but on standard therapy for the duration of the current study.

Patients with significant, unstable or uncontrolled acute or chronic diseases unrelated to SLE were excluded (i.e., cardiovascular, pulmonary, hematologic, gastrointestinal, hepatic, renal, neurological, malignancy or infectious diseases), as were those with an adverse event (AE) that would have placed the patient at undue risk in the belimumab open-label continuation studies. Patients were ineligible for entry into the TH or TC groups if during the screening period they had experienced a mild-moderate or severe flare as defined by the SELENA-SLEDAI Flare Index (SFI), received > 20 mg/day of prednisone (or equivalent) or received any new immunosuppressive/immunomodulatory agent, antimalarial or non-steroidal anti-inflammatory drug.

### Study endpoints and assessments

The primary endpoint was median time to any SFI flare (defined in Additional materials). Secondary endpoints were rate of any SFI flare, time to severe SFI flare and time to renal flare (defined in Additional materials). Additionally, evidence of a rebound in both the TH and LTD groups (defined as a SELENA-SLEDAI score that exceeded the baseline score in the original parent study at any time from baseline of the current study up to and including Week 24) and prednisone use were assessed. Efficacy assessments included the Physician Global Assessment (PGA; defined in Additional materials).

Safety assessments included monitoring of AEs, serious AEs (SAEs), AEs of special interest (AESI), haematological and clinical chemistry parameters, urinalysis, B cell markers and immunogenicity markers (anti-drug antibodies). AE or SAE grading was based on the Division of Microbiology and Infectious Diseases Adverse Event Severity Grade Tables.

Biomarker levels were monitored over the study period. These included immunoglobulins (IgG, IgM and IgA), anti-double-stranded deoxyribonucleic acid (anti-dsDNA), C3 and C4, and B cell subsets (CD19+, CD20+ and naïve CD19+/CD20+CD27−).

### Data analyses

All analyses were descriptive and performed on the intention-to-treat (ITT) population, defined as all patients that enrolled in the study, excluding screen failures. The probability of experiencing the first SFI flare and the median time to the first SFI flare were estimated using the Kaplan–Meier method. The primary efficacy analysis was a descriptive comparison of the median time to the first SFI flare.

The original enrolment target was 135 patients; however, owing to slow enrolment as a result of patients’ reluctance to take a belimumab TH and the limited availability of patients from ongoing belimumab studies, this target was revised to 71 patients (10 in the TH group, 26 in the TC group and 35 in the LTD group). This sample size was expected to provide adequate information to characterise time to SFI flare. The study was not controlled or randomised and was not powered to show a difference between the study groups.

Unless otherwise stated, the baseline was day 0 of the current study.

### Patient and public involvement

Patients or the public were not involved in the design, conduct, reporting or dissemination plans of our research.

## Results

### Study population

Of the 103 screened patients, 80 were enrolled and included in the ITT population: 12 (15.0%) in the TH group, 29 (36.3%) in the TC group and 39 (48.8%) in the LTD group (Table 1). Prior to week 52, 9 patients (11.3%) withdrew from the treatment/observation phase, 1 patient in the TH group moved to the maintenance phase due to condition worsening during treatment discontinuation, and a further 25 patients in the TH and TC groups entered the maintenance phase after completing the treatment/observation phase. Of these 26 patients, 9 (34.6%) completed the maintenance phase (Additional Table S1). Baseline demographics and disease characteristics are shown in Table 1. All patients in the TH and TC groups and the majority of the LTD group (61.5%) were Asian. The LTD group showed greater evidence of disease activity at baseline than the TH and TC groups, as expected based on the inclusion criteria (Table 1). In the LTD group, 30.8% of patients were not receiving prednisone at baseline, compared with 8.3% and 3.4% of patients in the TH and TC groups, respectively.

**Table 1:** Baseline demographics and clinical characteristics (ITT population)

	TH (***n*** = 12)	TC (***n*** = 29)	LTD (***n*** = 39)	Total (***N*** = 80)
**Country,** ***n*** **(%)**				
China	4 (33.3)	16 (55.2)	0	20 (25.0)
Japan	8 (66.7)	2 (6.9)	0	10 (12.5)
Korea	0	11 (37.9)	23 (59.0)	34 (42.5)
USA	0	0	16 (41.0)	16 (20.0)
**Sex,** ***n*** **(%)**				
Female	9 (75.0)	27 (93.1)	35 (89.7)	71 (88.8)
Male	3 (25.0)	2 (6.9)	4 (10.3)	9 (11.3)
**Mean (SD) age, years (at screening)**	38.1 (8.04)	40.6 (9.76)	38.9 (12.01)	39.4 (10.63)
**Race,** ***n*** **(%)**				
White/Caucasian/European heritage	0	0	9 (23.1)	9 (11.3)
Asian	12 (100)	29 (100)	24 (61.5)	65 (81.3)
African-American/Black African Ancestry	0	0	5 (12.8)	5 (6.3)
Native Hawaiian or other Pacific Islander	0	0	1 (2.6)	1 (1.3)
**Treatments,** ***n*** **(%)**				
Steroids (prednisone equivalent)^a^	11 (91.7)	28 (96.6)	25 (64.1)^b^	64 (80.0)
Antimalarials^c^	5 (41.7)	19 (65.5)	27 (69.2)	51 (63.8)
Immunosuppressants^d^	6 (50.0)	13 (44.8)	19 (48.7)	38 (47.5)
**Daily prednisone dose**^**e**^**, mg/day**				
Mean (SD)	7.5 (4.04)	5.7 (3.36)	3.7 (3.62)	5.0 (3.82)
Median (IQR)	6.8 (5.0, 10.0)	5.0 (2.5, 10.0)	2.5 (0.0, 7.5)	5.0 (1.3, 7.5)
Minimum, maximum	0.0, 15.0	0.0, 10.0	0.0, 10.0	0.0, 15.0
**By category,** ***n*** **(%)**				
0	1 (8.3)	1 (3.4)	12 (30.8)	14 (17.5)
> 0 to ≤ 7.5	6 (50.0)	20 (69.0)	21 (53.8)	47 (58.8)
> 7.5	5 (41.7)	8 (27.6)	6 (15.4)	19 (23.8)
**SELENA-SLEDAI category**^**f**^**,** ***n*** **(%)**				
≤ 3	11 (91.7)	22 (75.9)	16 (41.0)	49 (61.3)
> 3–9	1 (8.3)	7 (24.1)	22 (56.4)	30 (37.5)
≥ 10	0	0	1 (2.6)	1 (1.3)
**Mean (SD) SELENA-SLEDAI score**^**f**^	1.7 (1.67)	2.3 (1.46)	4.0 (2.41)	3.0 (2.21)
**SFI,** ***n*** **(%)**				
≥ 1 flare^g^	1 (8.3)	0	8 (20.5)	9 (11.3)
≥ 1 severe flare	0	0	1 (2.6)	1 (1.3)

*IQR* interquartile range, *ITT* intention-to-treat, *LTD* long-term discontinuation, *SD* standard deviation, *SELENA* Safety of Estrogens in Lupus Erythematosus National Assessment, *SFI* SELENA-SLEDAI Flare Index, *SLEDAI* SLE Disease Activity Index, *TC* treatment continuation, *TH* treatment holiday

^a^Steroid use was assessed in the 7 days prior to day 0 of the study

^b^Excludes 2 patients on prednisone prior to day 0 of this study, but who did not take prednisone in the 7 days prior to day 0

^c^Hydroxychloroquine and hydroxychloroquine sulphate

^d^Azathioprine, cyclosporine, leflunomide, methotrexate, mizoribine, mycophenolate mofetil, mycophenolic acid, tacrolimus and thalidomide

^e^Based on all days from the screening visit date of the current study up to and including day 0 of the current study

^f^Provided by post hoc analysis

^g^One patient’s baseline visit SFI flare result was collected at week 4 visit and was included in the count of patients with ≥ 1 flare at baseline

### Efficacy

**Fig. 2:**
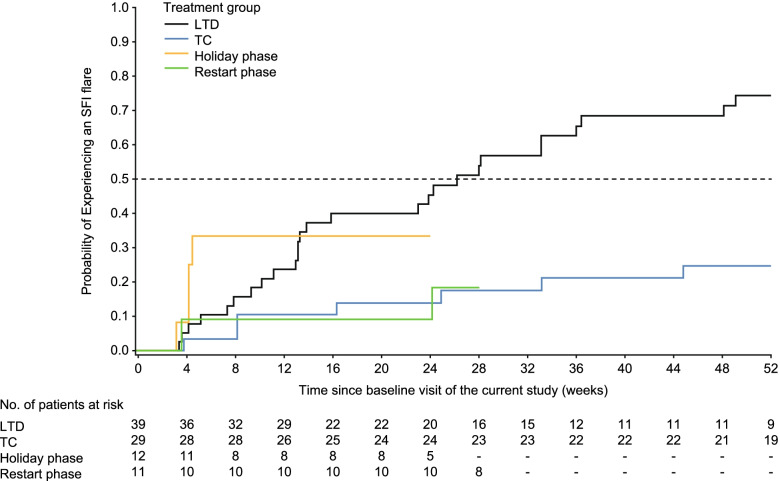
Probability of experiencing an SFI flare before week 52 (ITT population). Median survival time is the time at which the curve crosses 0.5; if the curve does not cross 0.5, the median is not defined; all data are presented relative to time 0; for the restart phase, time 0 is the time when the patient re-initiated belimumab treatment post-week 24 in the study. ITT, intention-to-treat; LTD, long-term discontinuation; SFI, SELENA-SLEDAI Flare Index; SELENA, Safety of Estrogens in Lupus Erythematosus National Assessment; TC, treatment continuation; TH, treatment holiday

The primary endpoint, median time to first SFI flare, was not evaluable in the TH and TC groups, as fewer than half of the patients in these groups flared. The median days (interquartile range [IQR]) to first SFI flare in the LTD group was 183.0 (91.0, 370.0). The probability of experiencing a first SFI flare to week 52 is shown in Fig. 2. In the TH group, 4 (33.3%) patients experienced SFI flares in the first 24 weeks, and 2 (18.2%) experienced SFI flares in the 28-week restart phase (Table 2). In total, 5 (41.7%) patients in the TH group, 9 (31.0%) in the TC group and 28 (71.8%) in the LTD group (the highest percentage of the 3 groups) experienced SFI flares to week 52 (Table 2).

**Table 2:** Efficacy outcomes (52-week study period; ITT population)

	TH (***n*** = 12)			TC (***n*** = 29)	LTD (***n*** = 39)
	24-week holiday phase (***n*** = 12)	28-week restart phase (***n*** = 11)	Overall 52-week period (***n*** = 12)		
Patients with an SFI flare, *n* (%)	4 (33.3)	2 (18.2)	5 (41.7)^a^	9 (31.0)	28 (71.8)
Time to first SFI flare, median (IQR) days^b^	- (30.0, -)	- (-, -)	- (30.0, -)^a^	- (365.0, -)	183.0 (91.0, 370.0)
Unadjusted rate of SFI flares/patient-year	1.0	0.3	0.6^a^	0.6	2.1
Number of SFI flares	5	2	7^a^	17	74
Patients with a severe SFI flare, *n* (%)	0	0	0	0	9 (23.1)
Patients with a renal flare^c^, *n* (%)	0	0	0	0	4 (10.3)
Daily prednisone use^d^, median (IQR) days					
> 7.5 mg/day and/or 25% increase vs baseline^e^	168.0 (168.0, 170.0)	196.0 (196.0, 196.0)	364.0 (364.0, 364.0)	364.0 (311.5, 371.0)	278.0 (133.0, 344.0)
≤ 7.5 mg/day and/or 25% decrease vs baseline^f^	166.0 (161.0, 168.0)	200.0 (196.0, 202.0)	364.0 (359.0, 364.0)	364.0 (364.0, 364.0)	362.0 (227.0, 365.0)

*IQR* interquartile range, *ITT* intention-to-treat, *LTD* long-term discontinuation, *SFI* SELENA-SLEDAI Flare Index, *SELENA* Safety of Estrogens in Lupus Erythematosus National Assessment, *SLEDAI* SLE Disease Activity Index, *TC* treatment continuation, *TH* treatment holiday

^a^Provided by post hoc analysis

^b^Estimated using the Kaplan–Meier method; statistics are missing when the number of events is too low to estimate the value

^c^Defined in Additional file 1

^d^Average daily prednisone dose at baseline is based on all days from the screening visit date of the current study up to and including day 0 of the current study

^e^TH: *n* = 6, TC: *n* = 12; LTD: *n* = 13

^f^TH: *n* = 6, TC: *n* = 19; LTD: *n* = 25

The unadjusted rate of SFI flares per patient-year was 1.0 during the holiday phase, 0.3 during the restart phase and 0.6 in the overall 52-week period in the TH group, and 0.6 in the TC group, in contrast to 2.1 in the LTD group (Table 2). The LTD group was the only group in which patients experienced severe SFI flares (*n* = 9, 23.1%) or renal flares (*n* = 4, 10.3%) (Table 2); therefore, the median time to first severe SFI or renal flare was not evaluable in the TH and TC groups. Among patients experiencing a severe SFI or renal flare in the LTD group, the median (IQR) study day of the first flare was day 162 (36.0, 225.0) and day 239 (224.5, 295.0), respectively.

No TH patients rebounded in the first 24 weeks, whilst 2 (6.9%) patients in the TC group and 2 (5.1%) patients in the LTD group rebounded (Table 3); all rebound patients had SELENA-SLEDAI score > 3 at the baseline.

**Table 3:** Mean parent study baseline SELENA-SLEDAI score and number of current study rebound patients (ITT population)

	TH (***n*** = 12)		TC (***n*** = 29)	LTD (***n*** = 39)
	24-week holiday phase (***n*** = 12)	28-week restart phase (***n*** = 11)		
Mean (SD) baseline SELENA-SLEDAI score in parent study	7.9 (4.19)	NA	9.2 (3.06)	10.2 (3.15)
Number of rebound patients in first 24 weeks of current study^a^, *n* (%)	0	NA	2 (6.9)	2 (5.1)

*ITT*, intention-to-treat; *LTD*, long-term discontinuation; *SELENA*, Safety of Estrogens in Lupus Erythematosus National Assessment; *SD*, standard deviation; *SLEDAI*, SLE Disease Activity Index; *TC*, treatment continuation; *TH*, treatment holiday

^a^SELENA-SLEDAI score exceeding parent study baseline anytime between baseline of the current study up to and including week 24

The proportion of patients with an increased use of prednisone to > 7.5 mg/day or a dose 25% higher than that at baseline was similar in the TH and TC groups (*n* = 6, 50.0%, and *n* = 12, 41.4%, respectively) and lower in the LTD group (*n* = 13, 33.3%) in the overall 52-week period. The median number of days on which prednisone use was > 7.5 mg/day and/or the dose had increased from baseline by 25% was similar in the TH and TC groups over the 52-week treatment period and lower in the LTD group (Table 2). The median number of days on which prednisone use was ≤ 7.5 mg/day and/or the dose had decreased from baseline by 25% was similar in all three groups, although there is some evidence of separation based on the IQR values (Table 2).

At week 52, there was little change in the median (IQR) PGA score from baseline in all three groups (TH, 0.00 [− 0.09, 0.15]; TC, − 0.03 [− 0.15, 0.03]; LTD, 0.00 [− 0.12, 0.27]).

Treatment comparisons between the LTD and the TC and TH groups should be interpreted with caution owing to the differences in baseline patient characteristics.

### Safety

During the 52-week study period, the incidence of AEs and SAEs was highest in the LTD group; there was only 1 SAE in the TH group, which occurred during the restart phase and was considered to be unrelated to the study treatment (Table 4). The most common AEs reported in each group were upper respiratory tract infection (*n* = 2, 16.7%) in the TH group during the holiday phase, and nasopharyngitis in the TH group during the restart phase (*n* = 4, 36.4%), the TC group (*n* = 5, 17.2%) and the LTD group (*n* = 10, 25.6%). Treatment-related AEs occurred in 2 (16.7%) patients in the TH group during the holiday phase, 1 (9.1%) patient in the TH group during the restart phase and 6 (20.7%) patients in the TC group (Table 4). The investigator considered there was a reasonable possibility that the AEs observed in the TH group during the holiday phase were related to belimumab, despite the patients not being on the treatment at the time. No AESI were reported in the TH group; the incidences of AESI were similar between the TC and LTD groups (Table 4). There were no withdrawals due to AEs and no deaths.

**Table 4:** Most common AEs by SOC^a^, all SAEs, AESI, and TRAEs (52-week study period; ITT population)

	TH (***n*** = 12)			TC (***n*** = 29)	LTD (***n*** = 39)
	24-week holiday phase (***n*** = 12)	28-week restart phase (***n*** = 11)	Overall 52-week period^a^ (***n*** = 12)		
**≥ 1 AE by SOC**^**b,c**^**,** ***n*** **(%)**	7 (58.3)	10 (90.9)	11 (91.7)	21 (72.4)	37 (94.9)
Infections and infestations	4 (33.3)	7 (63.6)	8 (66.7)	14 (48.3)	23 (59.0)
**≥ 1 SAE by preferred term,** ***n*** **(%)**	0	1 (9.1)	1 (8.3)	2 (6.9)	6 (15.4)
Lupus nephritis	0	0	0	0	2 (5.1)
Acute respiratory failure	0	0	0	0	1 (2.6)
Arthritis	0	0	0	1 (3.4)	0
Chronic obstructive pulmonary disease	0	0	0	0	1 (2.6)
Diarrhoea infectious	0	0	0	0	1 (2.6)
Generalised oedema	0	0	0	0	1 (2.6)
Lumbar vertebral fracture	0	1 (9.1)	1 (8.3)	0	0
Pericarditis lupus	0	0	0	0	1 (2.6)
Pneumonia pseudomonal	0	0	0	0	1 (2.6)
Respiratory failure	0	0	0	0	1 (2.6)
Sepsis	0	0	0	0	1 (2.6)
Upper respiratory tract infection	0	0	0	1 (3.4)	0
**AESI by category,** ***n*** **(%)**					
Malignancies	0	0	0	0	0
Post-infusion systemic reactions	0	0	0	1 (3.4)^d^	0
Infections of special interest^e^	0	0	0	1 (3.4)	3 (7.7)
Depression	0	0	0	1 (3.4)^f^	1 (2.6)^f^
Deaths	0	0	0	0	0
**TRAEs by system organ class,** ***n*** **(%)**					
Any event	2 (16.7)	1 (9.1)	3 (25.0)	6 (20.7)^g^	0
Infections and infestations	2 (16.7)	0	2 (16.7)	5 (17.2)	0
Respiratory, thoracic and mediastinal disorders	0	0	0	2 (6.9)	0
Gastrointestinal disorders	0	1 (9.1)	1 (8.3)	0	0
Skin and subcutaneous tissue disorders	0	0	0	1 (3.4)	0

*AE*, adverse event; *AESI*, AE of special interest; *ITT*, intention-to-treat; *LTD*, long-term discontinuation; *SAE*, serious AE; *SOC*, system organ class; *TC*, treatment continuation; *TH*, treatment holiday; *TRAE*, treatment-related AE

^a^Provided by post hoc analysis

^b^Reported in > 50% of patients in any treatment group

^c^Nasopharyngitis was the most common AE in all three treatment groups (LTD, TC and restart phase)

^d^Post-infusion systemic reactions per anaphylactic reaction customised MedDRA query broad search

^e^By preferred term, herpes simplex (TC, *n* = 1; LTD, *n* = 1), recurrent opportunistic herpes zoster (LTD, *n* = 1), serious sepsis (LTD, *n* = 1)

^f^Depression including mood disorders and anxiety

^g^> 1 event occurred in 1 patient

There were no grade 3/4 laboratory abnormalities in the TH group during the 52-week study period; in the other groups, the most common laboratory abnormalities were grade 3 protein/creatinine increase observed in 1 patient (3.4%) in the TC group and 3 patients (7.7%) in the LTD group, with grade 4 increase observed in 6 patients (15.4%) in the LTD group (Additional Table S2).

There were no positive immunogenicity responses during the 52-week study period.

### Biomarkers

At the baseline of the current study, median IgG levels were below parent study baseline and remained decreased over 52 weeks, for all groups, with a slight increase towards the end of the holiday phase that fell again following treatment restart (Additional Figure S1). The median levels of anti-dsDNA remained below (Additional Figure S2), and the median C3/C4 levels remained above (Additional Figure S3), parent study baseline over 52 weeks for all groups. B cell levels remained depleted in the TC group throughout the 52-week study period. In the TH and LTD groups, CD19, CD20 and naïve B cell counts started to return to pre-treatment levels after 16 weeks off treatment but rapidly decreased to minimal levels (median percent change around − 70% relative to parent study baseline) following treatment restart in the TH group (Additional Figure S4).

### Maintenance phase

The maintenance phase enrolled patients from the TH and TC groups only, and no clear trends were apparent from these data. No severe SFI flares were reported in any patients in either group, and no new safety signals were noted. Post hoc analysis showed that the most commonly reported (≥ 2 patients in either treatment group) non-serious AEs occurring in this phase were nasopharyngitis (2 patients in the TC group and 4 patients In the TH group) and upper respiratory tract infection (2 patients in the TC group). No SAEs were observed.

## Discussion

This open-label study is the first to examine the clinical impact of temporarily discontinuing belimumab treatment in patients with SLE and low disease activity. Over 52 weeks, the flare rate was similar in patients who discontinued belimumab for 24 weeks and then restarted treatment, and in those who received continuous belimumab throughout the study. However, the primary endpoint, the median time to first SFI flare, was not evaluable in the TH and TC groups as fewer than half of the patients in each group experienced SFI flares. Whilst 2 patients in the LTD group and 2 patients in the TC group rebounded, no patients in the TH group rebounded. Changes in prednisone use were similar in the TH and TC groups, whilst fewer patients in the LTD group reported increased use of prednisone to > 7.5 mg/day or a 25% increase in dose versus baseline. Overall, temporary, 24-week belimumab discontinuation in patients with SLE and low disease activity did not appear to increase the risk of SFI flares or rebound. Importantly, treatment restart after the 24-week holiday phase did not result in post-infusion systemic reactions or the development of anti-drug antibodies. No new safety concerns were identified during the study (including the maintenance phase), and AEs were aligned with the known belimumab safety profile.

Both the TH and TC groups included patients with low baseline disease activity; as such, low flare rates in these groups were expected [14]. The low SFI flare rate following belimumab discontinuation may be explained, in part, by a molar excess of belimumab relative to BLyS in circulation during the first 3 months of the holiday phase (i.e. the belimumab washout period) [15–17]. However, the likelihood of an SFI flare remained low throughout the 24-week phase, even after the 3-month belimumab washout. The data suggested a higher SFI flare rate during the holiday phase than the restart phase (1.0 vs 0.3 per patient-year) in the TH group, although the SFI flare rate over the 52-week study period was similar in the TH and TC groups (0.6 per patient-year). However, low patient numbers make meaningful between-group comparisons difficult. The unadjusted 52-week SFI flare rate in the LTD group was much higher than in the TH or TC groups (2.1 vs 0.6 or 0.6 per patient-year, respectively), likely based on the higher disease activity in the LTD group at baseline.

Previous studies have shown significant reductions in anti-dsDNA antibody titres and increases in C3 and C4 levels with belimumab treatment, as well as sustained reductions in CD20+ B cells [9, 10]. These serologic changes are thought to reflect the ability of belimumab to neutralise BLyS, which may restore apoptosis of autoimmune B cells, ultimately leading to reduced SLE activity [18–20]. As expected, based on belimumab’s mechanism of action, the two patient groups discontinuing belimumab saw increases in levels of CD19+, CD20+ and naïve (CD19+/CD20+/CD27−) B cells 16 weeks after stopping treatment, which were reduced again when treatment was restarted. Based on the current data, it is not clear whether these serologic changes would lead to clinical differences in flare rate, as the baseline flare rate in this study was low. Further studies will therefore be useful to fully determine the association between the serological and clinical effects of temporarily discontinuing belimumab in patients with SLE.

It is now well established that belimumab treatment is associated with a reduced need for steroid use in patients with SLE [9, 10]. In phase 3 belimumab studies, these effects were seen within 8 weeks of starting belimumab treatment and were sustained over the 52- or 76-week study periods [9, 10]. In the current study, increases in prednisone use (to > 7.5 mg/day or a dose 25% higher than that at baseline) were seen in similar proportions of patients in the TH and TC groups (baseline to week 52/exit), suggesting that temporary belimumab discontinuation does not impact the reduced need for prednisone seen upon belimumab initiation. Of interest, the number of days on which prednisone dose increased (reported for patients who were taking ≤ 7.5 or > 7.5 mg/day at baseline) was lowest in the LTD group, perhaps reflecting more stable steroid usage in this group, although this group had a higher percentage of patients not on prednisone, as well as on background therapies other than prednisone (antimalarials/immunosuppressants), at baseline than the TH and TC groups. Alternatively, the lower disease activity in the TH and TC groups may be associated with higher prednisone use than the LTD group. Due to small sample sizes, comparisons between the three groups should be interpreted with caution.

There are several study limitations. First, as patients had completed previous belimumab trials, recruitment was biassed toward those less likely to experience belimumab-related AEs, as patients experiencing AEs in parent studies that were considered to place them at undue risk would be excluded from the present study. Furthermore, the sample size was small due to recruitment difficulties. There were also clinically important differences in patient characteristics in all three study groups at the start of the study, limiting the ability to draw inferences. Additionally, patients were assigned to the study groups based on a joint decision by the patient and investigator, and the study had a short treatment holiday period (6 months). With these limitations considered, these data provide a basis on which further studies can be conducted to build a better understanding of the clinical effects of temporary belimumab discontinuation for patients who may require such a treatment break. Notably, the TH and TC groups contained only patients of Asian heritage; however, previous studies have shown that the benefit-risk profile of belimumab treatment in the Asian population is similar to that observed among patients in the global phase 3 studies [10, 12]. Therefore, the results from the current study can be expected to be similar in the general SLE population.

## Conclusions

Temporary discontinuation of belimumab does not appear to increase the risk of SFI flare or rebound over a 6-month period in patients who have achieved low disease activity with belimumab treatment. Rebound was similar in patients with any level of SLE disease activity, but there was a higher rate of flares in this group. However, the number of patients who temporarily discontinued treatment was small, as was the number of patients who continued treatment. As such, a meaningful comparison between the groups was difficult. There were no new safety findings in this study, and the results continue to support the favourable benefit-risk profile of belimumab in patients with SLE.

## Supplementary Information

**Additional file 1 **: **Endpoint definitions. Table S1.** Patient disposition in the treatment/observation and maintenance phases (ITT population). **Table S2.** Grade 3/4a laboratory abnormalities observed during the treatment/observation and treatment holiday phases (ITT population). **Figure S1.** IgG levels percentage change from parent study baseline by visit (ITT population). **Figure S2.** Anti-dsDNA antibody levels percentage change from parent study baseline by visit (ITT population). **Figure S3.** Percentage change from parent study baseline in complement levels by visit (ITT population). **Figure S4.** CD19+, CD20+ and naïve B-cell median percentage change from parent study baseline (ITT population).

## Data Availability

Anonymised individual patient data and study documents can be requested for further research from www.clinicalstudydatarequest.com.

## Abbreviations

AE: Adverse event
AESI: Adverse event of special interest
BLISS: Study of Belimumab in Subjects with SLE
BLyS: B-lymphocyte stimulator
C: Component
dsDNA: Double-stranded deoxyribonucleic acid
Ig: Immunoglobulin
IQR: Interquartile range
ITT: Intention-to-treat
IV: Intravenous
LTD: Long-term discontinuation
q4w: Every 4 weeks
OLE: Open-label extension
PGA: Physician’s Global Assessment
SAE: Serious AE
SD: Standard deviation
SELENA-SLEDAI: Safety of Estrogens in Lupus Erythematosus National Assessment-SLE Disease Activity Index
SFI: SELENA-SLEDAI Flare Index
SLE: Systemic lupus erythematosus
SOC: System organ class
TC: Treatment continuation
TH: Treatment holiday
TRAE: Treatment-related adverse event
VAS: Visual analogue scale

## References

[1] Grammer AC, Lipsky PE (2003). B cell abnormalities in systemic lupus erythematosus. Arthritis Res Ther.

[2] Manson JJ, Rahman A (2006). Systemic lupus erythematosus. Orphanet J Rare Dis.

[3] Kan HJ, Song X, Johnson BH, Bechtel B, O’Sullivan D, Molta CT (2013). Healthcare utilization and costs of systemic lupus erythematosus in Medicaid. Biomed Res Int.

[4] Conti F, Ceccarelli F, Perricone C, Leccese I, Massaro L, Pacucci VA (2016). The chronic damage in systemic lupus erythematosus is driven by flares, glucocorticoids and antiphospholipid antibodies: results from a monocentric cohort. Lupus..

[5] Fanouriakis A, Kostopoulou M, Alunno A, Aringer M, Bajema I, Boletis JN (2019). 2019 update of the EULAR recommendations for the management of systemic lupus erythematosus. Ann Rheum Dis.

[6] FDA (2020). Benlysta prescribing information.

[7] Baker KP, Edwards BM, Main SH, Choi GH, Wager RE, Halpern WG (2003). Generation and characterization of LymphoStat-B, a human monoclonal antibody that antagonizes the bioactivities of B lymphocyte stimulator. Arthritis Rheum.

[8] Hase H, Kanno Y, Kojima M, Hasegawa K, Sakurai D, Kojima H (2004). BAFF/BLyS can potentiate B-cell selection with the B-cell coreceptor complex. Blood..

[9] Furie R, Petri M, Zamani O, Cervera R, Wallace DJ, Tegzova D (2011). A phase III, randomized, placebo-controlled study of belimumab, a monoclonal antibody that inhibits B lymphocyte stimulator, in patients with systemic lupus erythematosus. Arthritis Rheum.

[10] Navarra SV, Guzman RM, Gallacher AE, Hall S, Levy RA, Jimenez RE (2011). Efficacy and safety of belimumab in patients with active systemic lupus erythematosus: a randomised, placebo-controlled, phase 3 trial. Lancet..

[11] Stohl W, Schwarting A, Okada M, Scheinberg M, Doria A, Hammer AE (2017). Efficacy and safety of subcutaneous belimumab in systemic lupus erythematosus: a fifty-two-week randomized, double-blind, placebo-controlled study. Arthritis Rheumatol.

[12] Zhang F, Bae SC, Bass D, Chu M, Egginton S, Gordon D (2018). A pivotal phase III, randomised, placebo-controlled study of belimumab in patients with systemic lupus erythematosus located in China, Japan and South Korea. Ann Rheum Dis.

[13] Furer V, Zisman D, Pokroy-Shapira E, Molad Y, Elkayam O, Paran D (2016). Systemic lupus erythematosus exacerbation following cessation of belimumab treatment. Scand J Rheumatol.

[14] Tselios K, Gladman DD, Touma Z, Su J, Anderson N, Urowitz MB (2019). Clinical remission and low disease activity outcomes over 10 years in systemic lupus erythematosus. Arthritis Care Res (Hoboken).

[15] Furie R, Stohl W, Ginzler EM, Becker M, Mishra N, Chatham W (2008). Biologic activity and safety of belimumab, a neutralizing anti-B-lymphocyte stimulator (BLyS) monoclonal antibody: a phase I trial in patients with systemic lupus erythematosus. Arthritis Res Ther.

[16] Struemper H, Chen C, Cai W (2013). Population pharmacokinetics of belimumab following intravenous administration in patients with systemic lupus erythematosus. J Clin Pharmacol.

[17] Shida Y, Takahashi N, Sakamoto T, Ino H, Endo A, Hirama T (2014). The pharmacokinetics and safety profiles of belimumab after single subcutaneous and intravenous doses in healthy Japanese volunteers. J Clin Pharm Ther.

[18] Carter RH, Zhao H, Liu X, Pelletier M, Chatham W, Kimberly R (2005). Expression and occupancy of BAFF-R on B cells in systemic lupus erythematosus. Arthritis Rheum.

[19] Petri M, Stohl W, Chatham W, McCune WJ, Chevrier M, Ryel J (2008). Association of plasma B lymphocyte stimulator levels and disease activity in systemic lupus erythematosus. Arthritis Rheum.

[20] Mackay F, Schneider P (2009). Cracking the BAFF code. Nat Rev Immunol.

