# Supplementary material for: The effect of 24-week belimumab treatment withdrawal followed by treatment restart in patients with SLE: an open-label, non-randomised 52-week study
Source: Arthritis Res Ther. 2022 Feb 16;24:46. doi: 10.1186/s13075-022-02723-y (PMC8848800; doi:10.1186/s13075-022-02723-y)
Supplement: Supplementary file 1 — Additional file 1 : Endpoint definitions. Table S1. Patient disposition in the treatment/observation and maintenance phases (ITT population). Table S2. Grade 3/4a laboratory abnormalities observed during the treatment/observation and treatment holiday phases (ITT population). Figure S1. IgG levels percentage change from parent study baseline by visit (ITT population). Figure S2. Anti-dsDNA antibody levels percentage change from parent study baseline by visit (ITT population). Figure S3. Percentage change from parent study baseline in complement levels by visit (ITT population). Figure S4. CD19+, CD20+ and naïve B-cell median percentage change from parent study baseline (ITT population). [file 13075_2022_2723_MOESM1_ESM.docx]

**Additional materials**

**Endpoint definitions:**

Safety of Estrogens in Lupus Erythematosus National Assessment-Systemic Lupus Erythematosus Disease Activity Index (SELENA-SLEDAI) flare index (SFI) flare: SLE flares were defined as a mild/moderate or severe, according to the modified SFI (excluded severe flares from the SELENA-SLEDAI flare assessment that were triggered only by an increase in SELENA-SLEDAI score to >12).

Renal flare: a reproducible increase in 24-h urine protein equivalent levels to: i) >1 g if the current study baseline value was <0.2 g; ii) >2g if the baseline value was 0.2–1 g; or iii) more than twice the value at baseline if the baseline value was >1 g.

Physician Global Assessment (PGA): a 0─10 cm visual analogue scale (VAS), anchored at 0 (none) and 3 (severe), with intermediate lines at 1 (mild), and 2 (moderate). Each PGA measurement was transformed linearly (x3/10) to obtain a value between 0.00 and 3.00. The same primary investigator or same sub-investigator evaluated and scored the PGA for the patient each time, unless agreed otherwise with the GSK medical monitor.

**Additional Table S1.** Patient disposition in the treatment/observation and maintenance phases (ITT population)

|  | **Patients, n (%)** | | | |
| --- | --- | --- | --- | --- |
|  | **TH (n=12)** | **TC (n=29)** | **LTD (n=39)** | **All patients (N=80)** |
| **Completed treatment/ observation phase** | 11 (91.7) | 27 (93.1) | 33 (84.6) | 71 (88.8) |
| **Withdrawn prior to Week 52** | 1 (8.3) | 2 (6.9) | 6 (15.4) | 9 (11.3) |
| Physician decision | 0 (0) | 0 (0) | 1 (2.6) | 1 (1.3) |
| Protocol deviation | 0 (0) | 2 (6.9) | 1 (2.6) | 3 (3.8) |
| Study terminated by sponsor | 1 (8.3) | 0 (0) | 0 (0) | 1 (1.3) |
| Withdrawal by patient | 0 (0) | 0 (0) | 4 (10.3) | 4 (5.0) |
| **Moved to maintenance**^a^ | 1 (8.3) | NA | NA | 1 (1.3) |
| **Entered maintenance phase** | 10 (83.3) | 16 (55.2) | NA | 26 (32.5) |
| **Completed maintenance phase**^b, c^ | 3 (30.0) | 6 (37.5) | NA | 9 (34.6) |
| **Withdrawn prior to end of maintenance phase**^c^ | 7 (70.0) | 10 (62.5) | NA | 17 (65.4) |
| Study terminated by sponsor | 7 (70.0) | 10 (62.5) | NA | 17 (65.4) |

^a^One patient was moved from the TH phase earlier than Week 24 due to worsening of SLE; ^b^patients were considered to have completed the maintenance phase if they discontinued study agent when belimumab became commercially available in their country; ^c^percentages were calculated relative to the number of patients who entered the maintenance phase.

ITT, intention-to-treat; LTD, long-term discontinuation; TC, treatment continuation; TH, treatment holiday

**Additional Table S2.** Grade 3/4^a^ laboratory abnormalities observed during the treatment/observation and treatment holiday phases (ITT population)

| **Parameter (unit), n (%)** | **TH** | | **TC (n=29)** | **LTD (n=39)** |
| --- | --- | --- | --- | --- |
|  | **24-week holiday phase (n=12)** | **28-week restart phase (n=11)** |  |  |
| **Neutrophils (10^9^ L)**  Grade 3  Grade 4 | 0 (0)  0 (0) | 0 (0)  0 (0) | 1 (3.4)  0 (0) | 1 (2.6)  1 (2.6) |
| **Leukocytes (10^9^ L)**  Grade 3  Grade 4 | 0 (0)  0 (0) | 0 (0)  0 (0) | 0 (0)  0 (0) | 1 (2.6)  0 (0) |
| **Albumin (g/L)**  Grade 3  Grade 4 | 0 (0)  0 (0) | 0 (0)  0 (0) | 0 (0)  0 (0) | 1 (2.6)  0 (0) |
| **Protein/creatinine (g/g)**  Grade 3  Grade 4 | 0 (0)  0 (0) | 0 (0)  0 (0) | 1 (3.4)  0 (0) | 3 (7.7)  6 (15.4) |
| **IgG (g/L)**  Grade 3  Grade 4 | 0 (0)  0 (0) | 0 (0)  0 (0) | 0 (0)  0 (0) | 1 (2.6)  0 (0) |

^a^Toxicity grading was based on the Division of Microbiology and Infectious Diseases Adult Toxicity Tables, 2001.

ITT, intention-to-treat; LTD, long-term discontinuation; TC, treatment continuation; TH, treatment holiday

**Additional Figure S1.** IgG levels percentage change from parent study baseline by visit (ITT population)


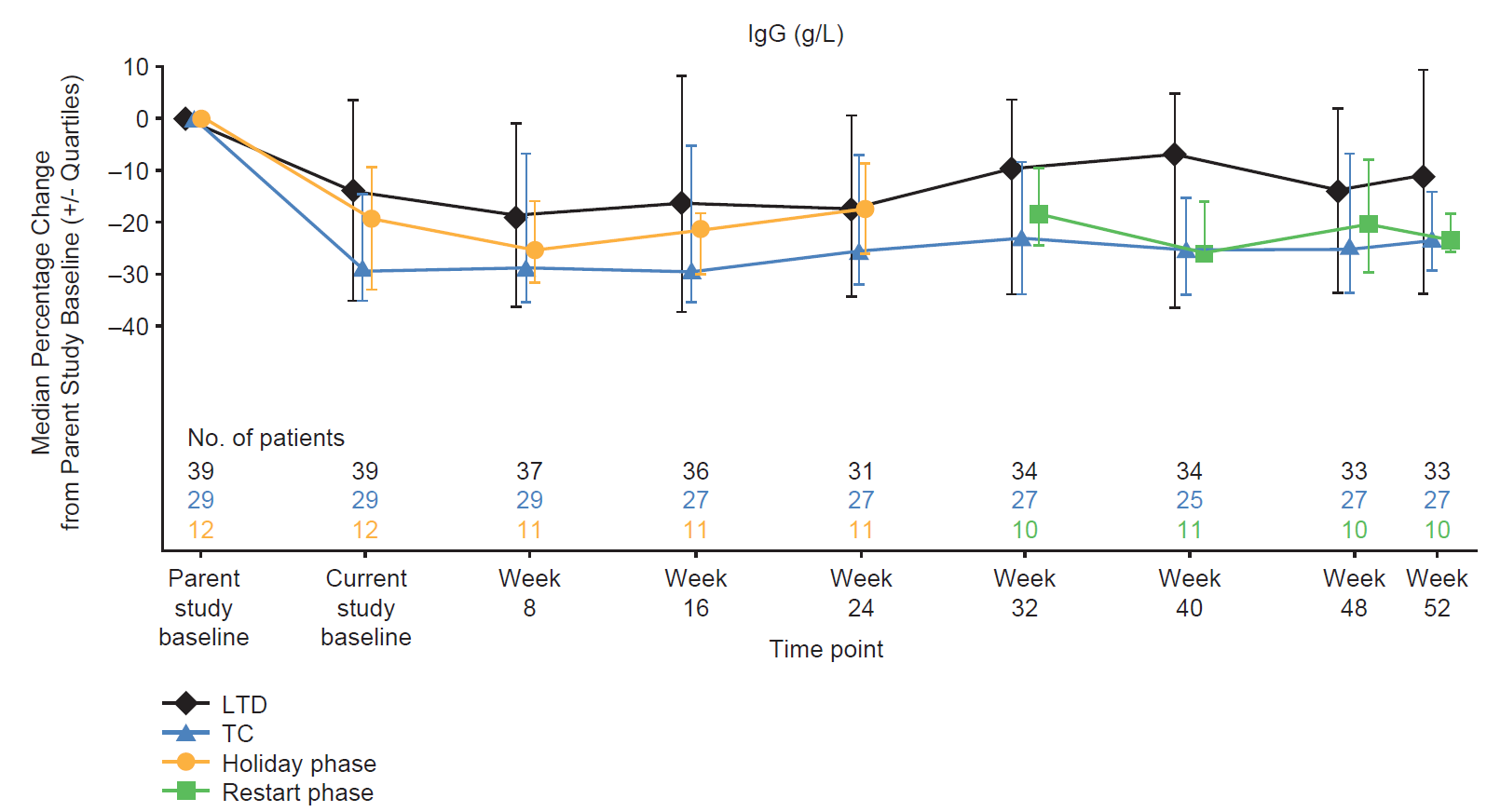


The median (IQR) IgG levels (g/L) at parent study baseline were: LTD 13.5 (10.6, 17.8); TC 14.3 (11.4, 19.5): holiday phase 15.0 (12.4, 17.2). Normal IgG range: 6.94–16.18 g/L.

ITT, intention-to-treat; IQR, interquartile range; LTD, long-term discontinuation; TC, treatment continuation

**Additional Figure S2.** Anti-dsDNA antibody levels percentage change from parent study baseline by visit (ITT population)


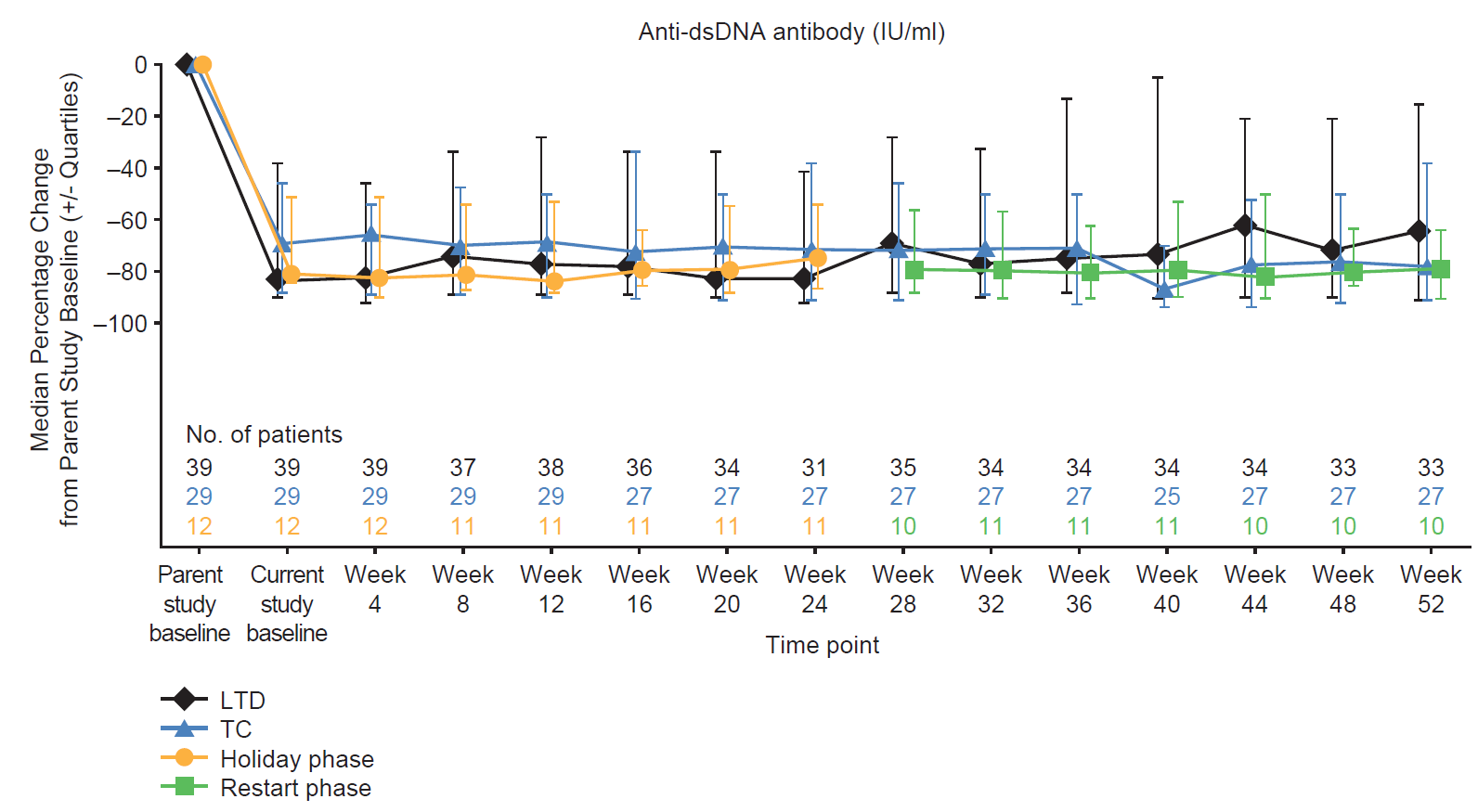


The median (IQR) anti-dsDNA antibody levels (IU/ml) at parent study baseline were: LTD 112.0 (39.0, 201.0); TC 135.0 (47.0, 355.0); holiday phase 137.0 (77.5, 275.0). Normal anti-dsDNA range: ≤30 IU/ml.

ITT, intention-to-treat; IQR, interquartile range; LTD, long-term discontinuation; TC, treatment continuation

**Additional Figure S3.** Percentage change from parent study baseline in complement levels by visit (ITT population)


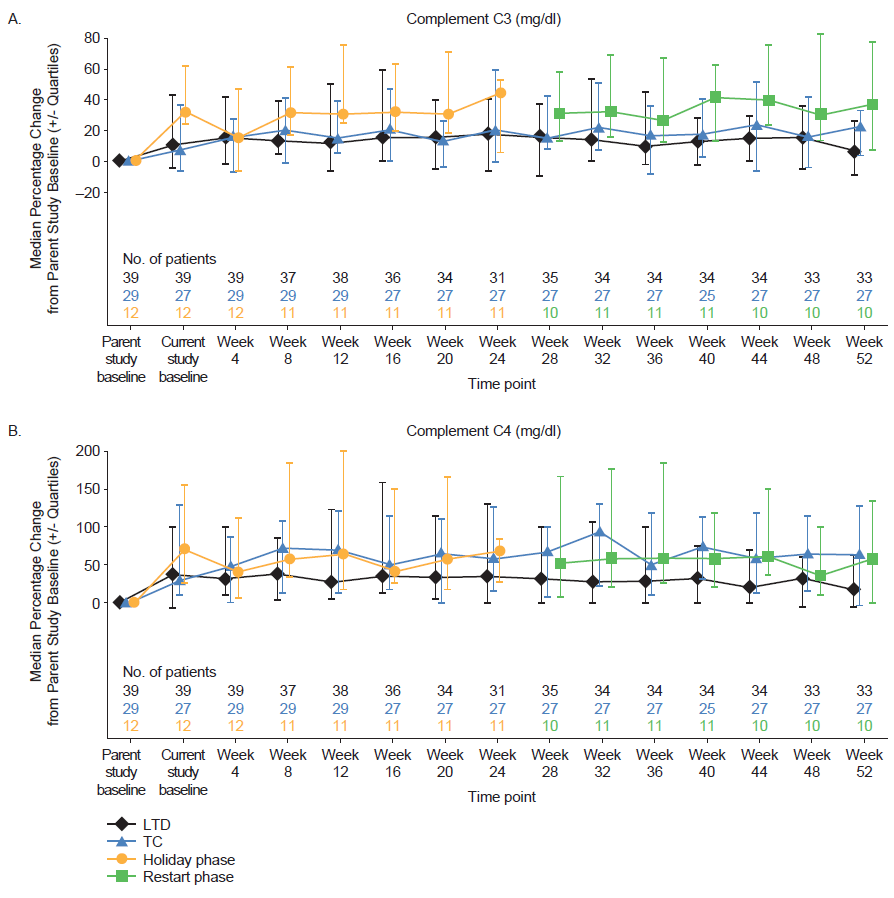


The median (IQR) C3 levels (mg/dl) at parent study baseline were: LTD 81.0 (60.0, 102.0); TC 78.0 (68.0, 97.0); holiday phase 82.0 (67.0, 97.0). The median (IQR) C4 levels (mg/dl) at parent study baseline were: LTD 13.0 (7.0, 19.0); TC 14.0 (8.0, 19.0); holiday phase 12.0 (7.0, 18.0). Normal C3 range: 90–180 mg/dl; normal C4 range: 10–40 mg/dl.

ITT, intention-to-treat; IQR, interquartile range; LTD, long-term discontinuation; TC, treatment continuation

**Additional Figure S4.** CD19+, CD20+ and naïve B-cell median percentage change from parent study baseline (ITT population)


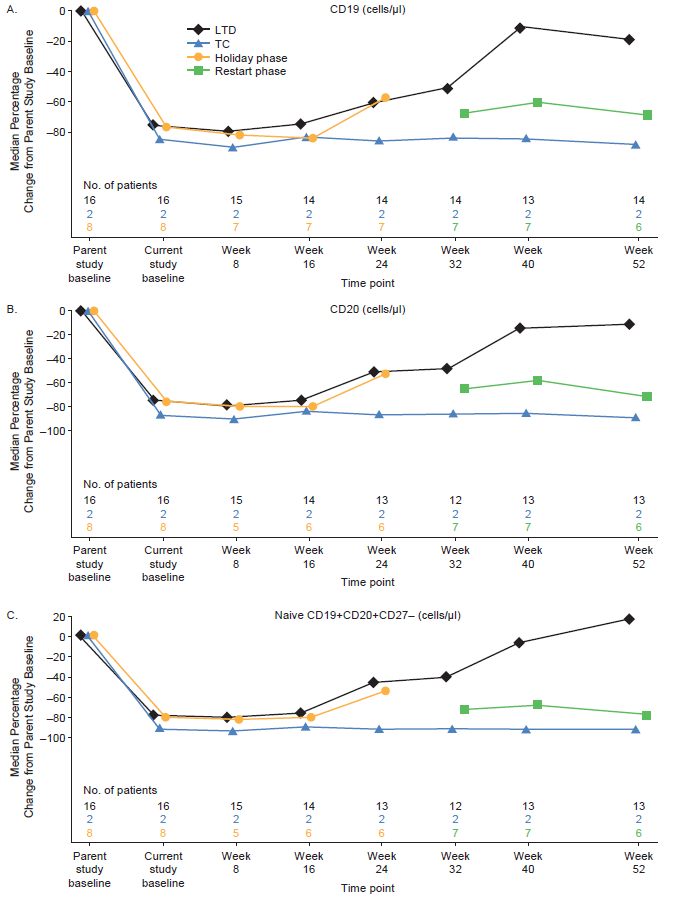


The median (IQR) CD19 levels (cells/µl) at parent study baseline were: LTD 91.0 (70.0, 211.0); TC 172.0 (100.0, 244.0); holiday phase 93.0 (50.0, 159.0). The median (IQR) CD20 levels (cells/µl) at parent study baseline were: LTD 90.0 (66.0, 208.5); TC 163.0 (87.0, 239.0); holiday phase 91.5 (48.0, 154.0). The median (IQR) naïve CD19+CD20+CD27− levels (cells/µl) at parent study baseline were: LTD 72.5 (56.0, 185.5); TC 140.0 (51.0, 229.0); holiday phase 82.5 (29.5, 147.5).

Quartile bars were omitted to avoid distortion caused by wide bars due to low patient numbers.

ITT, intention-to-treat; IQR, interquartile range; LTD, long-term discontinuation; TC, treatment continuation
